# Attenuation of acute stroke injury in rat brain by minocycline promotes blood–brain barrier remodeling and alternative microglia/macrophage activation during recovery

**DOI:** 10.1186/s12974-015-0245-4

**Published:** 2015-02-10

**Authors:** Yirong Yang, Victor M Salayandia, Jeffrey F Thompson, Lisa Y Yang, Eduardo Y Estrada, Yi Yang

**Affiliations:** Department of Neurology, University of New Mexico Health Sciences Center, 1 University of New Mexico, Albuquerque, NM 87131 USA; College of Pharmacy, University of New Mexico Health Sciences Center, 1 University of New Mexico, Albuquerque, NM 87131 USA

**Keywords:** Minocycline, MRI, stroke recovery, neurovascular remodeling, tight junction proteins, microglia/macrophage, inflammatory phenotypes

## Abstract

**Background:**

Minocycline reduces reperfusion injury by inhibiting matrix metalloproteinases (MMPs) and microglia activity after cerebral ischemia. Prior studies of minocycline investigated short-term neuroprotective effects during subacute stage of stroke; however, the late effects of minocycline against early reperfusion injury on neurovascular remodeling are less well studied. We have shown that spontaneous angiogenesis vessels in ischemic brain regions have high blood–brain barrier (BBB) permeability due to lack of major tight junction proteins (TJPs) in endothelial cells at three weeks. In the present study, we longitudinally investigated neurological outcome, neurovascular remodeling and microglia/macrophage alternative activation after spontaneous and minocycline-induced stroke recovery.

**Methods:**

Adult spontaneously hypertensive rats had a 90 minute transient middle cerebral artery occlusion. At the onset of reperfusion they received a single dose of minocycline (3 mg/kg intravenously) or a vehicle. They were studied at multiple time points up to four weeks with magnetic resonance imaging (MRI), immunohistochemistry and biochemistry.

**Results:**

Minocycline significantly reduced the infarct size and prevented tissue loss in the ischemic hemispheres compared to vehicle-treated rats from two to four weeks as measured with MRI. Cerebral blood flow measured with arterial spin labeling (ASL) showed that minocycline improved perfusion. Dynamic contrast-enhanced MRI indicated that minocycline reduced BBB permeability accompanied with higher levels of TJPs measured with Western blot. Increased MMP-2 and −3 were detected at four weeks. Active microglia/macrophage, surrounding and within the peri-infarct areas, expressed YM1, a marker of M2 microglia/macrophage activation, at four weeks. These microglia/macrophage expressed both pro-inflammatory factors tumor necrosis factors-α (TNF-α) and interleukin-1β (IL-1β) and anti-inflammatory factors transforming growth factor-β (TGF-β) and interleukin-10 (IL-10). Treatment with minocycline significantly reduced levels of TNF-α and IL-1β, and increased levels of TGF-β, IL-10 and YM1.

**Conclusions:**

Early minocycline treatment against reperfusion injury significantly promotes neurovascular remodeling during stroke recovery by reducing brain tissue loss, enhancing TJP expression in ischemic brains and facilitating neuroprotective phenotype alternative activation of microglia/macrophages.

**Electronic supplementary material:**

The online version of this article (doi:10.1186/s12974-015-0245-4) contains supplementary material, which is available to authorized users.

## Background

Matrix metalloproteinases (MMPs) are induced in the ischemic brain and contribute to cell death and blood–brain barrier (BBB) disruption at an early stage after stroke. Recent data indicate that the use of MMP inhibitors might lead to new therapies for acute cerebral ischemia-induced brain injury. Earlier, we administered synthetic MMP inhibitors prior to middle cerebral artery occlusion (MCAO) in rats, which demonstrated the proof-of-principle of improvement, but has limited clinical relevance [1-3]. Minocycline, a tetracycline derivative with MMP and inflammation inhibitory effects, has been shown to reduce reperfusion injury in animal models of cerebral ischemia and human stroke [4-9]. The neuroprotective effect of minocycline is associated with its ability to interfere with MMP activity including gelatin proteolytic activation (MMP-2 and −9); minocycline reduces brain MMP-9 levels in response to microvessel damage and inflammation during the early stage after stroke [4,5,7,8,10]. We have demonstrated that minocycline alone or along with normobaric hyperoxia at reperfusion onset effectively reduces infarct size and disruption of BBB at 48 hours after transient focal cerebral ischemia [11]. Our data also show that a profound reduction in MMP-9 level was observed for minocycline therapy compared to a vehicle at 48 hours. Recently, we showed that treatment with synthetic MMP inhibitor GM6001 prior to MCAO reduces tissue loss and facilitates angiogenesis in ischemic hemispheres during stroke recovery [3]. Therefore, we hypothesized that early treatment with minocycline which can reduce reperfusion injury could also facilitate recovery with BBB remodeling after stroke.

Minocycline is also a potent inhibitor for inflammatory responses [4,12], one of the important early contributors to brain injury after a stroke. Microglia, the brain’s resident immune cells, are activated in response to injury and orchestrate the brain’s inflammatory response [13]. Activated microglia have been the target of experimental and clinical studies focusing on neuroinflammation after focal cerebral ischemia [14,15]. Recent studies show that treatment with minocycline after reperfusion for between five and seven days significantly reduced microglial activation, which improved motor function during stroke recovery [16,17]. Furthermore, microglia can assume different activated phenotypes depending on the activating stimulus [18,19]. The microglial immunophenotype changes over time from pro-inflammatory to anti-inflammatory after ischemic stroke. These two distinct processes are denoted M1 for the early pro-inflammatory phase, and M2 for the later anti-inflammatory and repair phase. There is evidence that, at later times during recovery, microglia in anti-inflammatory phenotypes play a key role in the promotion of neurovascular remodeling through the release of growth-related proteins and cytokines from peripheral and resident immune cells [13,18-21]. We proposed that the spontaneous microglial immunophenotype changes after stroke can be influenced by early minocycline exposure [22,23].

In this study, minocycline was administrated at an early stage of reperfusion when MMP-2 and −9 induce BBB opening and inflammation [1,2,6,24]. We used MRI, histological and biochemical methods to monitor the time course of neurovascular remodeling in response to spontaneous and therapy-induced stroke recovery. We also studied the pro- and anti-inflammatory cytokines expressed by microglia/macrophages to characterize the functional alternative activation in response to minocycline treatment.

## Materials and methods

### Middle cerebral artery occlusion with reperfusion

The study was approved by the University of New Mexico Animal Care Committee (13-101061-HSC) and conformed to the National Institutes of Health guidelines for use of animals in research. A total of 60 male spontaneously hypertensive rats (SHR, 280 to 300 g in body weight. Harlan, Indianapolis, IN, USA) were subjected to 90 minutes of MCAO with reperfusion for 24 and 48 hours, and one, two, three and four weeks [2]. For the minocycline treatment experiments, groups studied were minocycline (n = 15) and the vehicle (n = 15), while rats in each group were followed up on for four weeks of reperfusion. The rat numbers did not reflect the rats which died around 12 to 17 days after reperfusion during the long-term studies (approximately 33% in control group and 11% in treated group) [3]. A single dose of minocycline (3 mg/kg, Sigma-Aldrich, St. Louis, MO, USA) [25] or the vehicle were administered to rats via the right femoral vein immediately after reperfusion onset. We chose the single dose of minocycline based on our previous studies [2,3,11,26], in which a single dose of MMP inhibitors prior to MCAO or minocycline at reperfusion onset was administered. Minocycline was dissolved in dimethyl sulfoxide (DMSO, Sigma-Aldrich, St. Louis, MO, USA) and diluted in 25% solutol (BASF, Florham Park, NJ, USA) to reduce the concentration of DMSO to less than 10% [11]. Control rats had the same dose of the solutol with DMSO, which are referred to in the present report as ‘vehicle treated’.

### Magnetic resonance imaging

Multimodal magnetic resonance imaging (MRI) of the rat, including relaxation time imaging, diffusion imaging, perfusion imaging and dynamic permeability imaging, was temporally conducted after 48 hours, and one, two and four weeks of reperfusion. The rat was placed in a dedicated holder, and positioned in the isocenter of a 4.7-Tesla MRI scanner (Bruker Biospin), which was equipped with a 40-cm bore, a 660 mT/m (rise time within 120 μs) gradient and shim systems (Bruker Biospin MRI, Billerica, MA, USA). To obtain good signal-to-noise ratio, a small bore linear RF coil (inner diameter = 72 mm) and a single tuned surface coil (RAPID Biomedical, Rimpar, Germany) were employed for signal excitation and detection, respectively [3,27,28]. During MRI, the rats were anaesthetized with 2.5% isoflurane (Phonenix, Clipper Distributing Company, St. Joseph, MO, USA) by mechanical ventilation. Respiration and heart rate were monitored during MRI measurements, and body temperature was maintained at 37.0 ± 0.5°C.

T2-weighted images were acquired with a fast spin-echo sequence (rapid acquisition with relaxation enhancement (RARE)) (Repetition Time (TR)/Echo Time (TE) = 5,000 ms/56 ms, Field of View (FOV) = 4 cm × 4 cm, slice thickness = 1 mm, inter-slice distance = 1.1 mm, number of slices = 12, matrix = 256 × 256, number of average = 3). Infarct area and peri-infarct area were manually delineated from T2 images. The delineated areas were used as the reference for all the other parametric images. The same slice location was prescribed for all the subsequent MRI protocols.

Multi-slice, multi-shot, diffusion-weighted echo-planar imaging (EPI) (TR/TE = 3,800 ms/38 ms; b-values = 600 and 1,900 s/mm2 in 30 directions; FOV = 4 cm × 4 cm, slice thickness = 1 mm, matrix = 256 × 256) was performed to assess tissue architecture. Quantitative apparent diffusion coefficient (ADC) maps were calculated on a voxel-wise basis, with a linear least-squares fit on the logarithm of the signal intensity versus the b-value for each diffusion direction. Based on the ADC maps, the eigenvalues of the diffusion tensor, ADC and fractional anisotropy (FA) maps were generated using ParaVision 5.1 (Bruker Biospin MRI, Bellerica, MA, USA).

Cerebral blood flow (CBF) was measured using the arterial spin labeling (ASL) method. The sequence: flow-sensitive alternating inversion recovery rapid acquisition with relaxation enhancement (FAIR-RARE) was used to implement ASL with parameters: TE/TR = 46 ms/16,000 ms, FOV = 4 cm × 4 cm, slice thickness = 1 mm, number of slices = 1, matrix = 128 × 128. The perfusion map was calculated using the ASL_Perfusion_Processing macro in ParaVision 5.1 (Bruker Biospin MRI). The principle is as follows: inversion recovery data from the imaging slice are acquired after selective inversion of the slice, and after inversion of the slice including the surrounding tissue, containing the supplying arteries. The difference of the inverse of the apparent T1 images then yields a measure of the CBF.

To non-invasively evaluate BBB permeability, we applied dynamic contrast-enhanced (DCE)-MRI and graphical analysis of the resultant image data [29]. The contrast agent Gd-DTPA (Magnevist, Bayer Healthcare Pharmaceuticals Inc., Wayne, NJ) at dose of 0.1 mM/kg was injected into the femoral vein. DCE-MRI was performed using a transverse fast T1 mapping that consisted of obtaining pre-contrast (three sequences) and post-contrast (16 sequences) images up to 45 minutes after the contrast injection. The details of pulse sequence T1_EPI for T1 mapping are: FOV = 4 cm × 4 cm, slice thickness = 1.5 mm, slice gap = 0, matrix size = 128 × 128, TR/TE =10,000 ms/8.3 ms, number of segments = 4, number of average = 1, total scan time = 2 m 40 s 0 ms. The T1 map was reconstructed with the t1epia fitting function in the Bruker ParaVision Image Sequence Analysis (ISA) tool. Previous research [30] have demonstrated that the blood-to-tissue transfer or influx constant, *K*_i_, could be obtained by graphical analysis of a timed series of tissue and arterial concentrations of contrast agent. Since the contrast agent concentration is proportional to changes of 1/T1(Δ(1/T1(t))), the map of *K*_i_ was constructed from repeated estimates of Δ(1/T1(t)). An in-house computer program in MATLAB (Mathworks, Massachusetts, USA), which implemented the above principle, was used to generate the *K*_i_ map.

### Immunohistochemistry

A total of 10 μm sections from rat brains fixed with 2% paraformaldehyde, 0.1 M sodium periodate and 0.075 M lysine in 100 mM phosphate buffer at pH 7.3 (PLP) were used for immunohistochemical analysis. Primary antibodies and dilutions used in immunohistochemistry (IHC) were: ionized calcium biding adapter molecule 1 (Iba-1) (1:400, Wako Chemicals USA, Inc. Richmond, VA, USA), tumor necrosis factors-α (TNF-α) (1:300, Abcam, Cambridge, MA, USA), interleukin-1β (IL-1β) (1:500, Abcam, Cambridge, MA, USA), interleukin-10 (IL-10) (1:1,000 , Abcam, Cambridge, MA, USA), transforming growth factor-β (TGF-β) (1:200 , Abcam, Cambridge, MA, USA), chitinase-like 3 (YM1) (1:100, StemCell Technologies, Vancouver, Canada), integrin alpha M (OX-42, or CD 11b) (1:100, Accurate Chemical & Scientific Corporation, Westbury, NY, USA), rat endothelial cell antigen-1 (RECA-1) (1:300, Abcam, Cambridge, MA, USA), and beta-type platelet-derived growth factor receptor (PDGFR-β) (1:100, Cell Signaling Technology, Danvers, MA, USA).

For immunofluorescence, brain sections were treated with acetone and blocked with 5% normal serum. Primary antibodies were incubated for 48 hours at 4°C. Sections were incubated for 90 minutes at 25°C with secondary antibodies conjugated with fluorescein isothiocyanate (FITC) or Cy-3 (Jackson Immuno-Research, West Grove, PA, USA). 4′-6-diamidino-2-phenylindole (DAPI) (Molecular Probes, Eugene, OR, USA) was used to label cell nuclei. Immunohistochemistry (IHC) negative controls were incubated without the primary antibody or with normal (non-immune) immunoglobulin Gs (IgGs) (Jackson Immuno-Research, West Grove, PA, USA) and no specific immunolabeling was detected.

All IHC slides were viewed on an Olympus BX-51 bright field and fluorescence microscope (Olympus America Inc. Center Valley, PA, USA). Dual or triple immunofluorescence slides were also imaged confocally to verify co-labeling (Zeiss LSM 510, Carl Zeiss Microimaging, Thornwood, NY, USA).

### Western blots

Western blots were performed to determine protein levels in ischemic white matter. Proteins were extracted in radioimmunoprecipitation assay (RIPA) buffer from ischemic and nonischemic hemispheres. A total of 50 μg of total proteins were separated on 4% to 20% gradient gels (Bio-Rad Laboratories, Hercules, CA, USA). The proteins were transferred to polyvinylidene fluoride (PVDF) membranes. The membranes were then incubated with primary antibodies: claudin-5 (1:500, Life Technologies, Grand Island, NY, USA), occluding (1:500, Life Technologies, Grand Island, NY, USA), zona occluden-1 (ZO-1) (1:500, Life Technologies, Grand Island, NY, USA), MMP-3 (1:1,000, Epitomics, Burlingame, CA, USA), TNF-α (1:500, Abcam), IL-1β (1:500, Abcam, Cambridge, MA, USA), IL-10 (1:1,000, Abcam, Cambridge, MA, USA), TGF-β (1:500, Abcam, Cambridge, MA, USA), YM1 (1:400, StemCell Technologies, Vancouver, Canada). The membranes were incubated with the respective secondary antibodies and blots were developed using the West Pico Detection System (Thermo Scientific, Rockford, IL USA). Protein bands were visualized on X-ray film. Semi-quantitation of target protein intensities was done with the use of ImageJ (National Institutes of Health, Bethesda, MD, USA), and actin (Sigma-Aldrich, St. Louis, MO, USA) immunoblots on the same PVDF membranes that were used to normalize protein loading and transfer. The results are reported as normalized band intensity for quantifying relative protein expression.

### Gelatin zymography

MMP-2 and −9 in brain tissue proteins extracted in RIPA buffer were analyzed by gelatin zymography. Briefly, 50 μg of total protein were electrophoretically separated on 10% SDS-polyacrylamide gels co-polymerized with gelatin (Sigma-Aldrich, St. Louis, MO, USA). Gels were then incubated for 90 hours at 37°C with a developing buffer before they were stained with Brilliant Blue R (Sigma-Aldrich, St. Louis, MO, USA) for 30 minutes. Gels were destained until clear bands of gelatinolytic activity appeared on a dark blue background. The gels were dried and scanned for densitometry (Alpha Imager™ 2200; Alpha Innotech, San Leandro, CA, USA). Supernatants of human fibrosarcoma HT-1080 cells were used as gelatinase standards for MMP-2 and −9.

### Quantification of Iba-1 fluorescence intensity and co-localization of cytokines with Iba-1

In order to evaluate the effect of minocycline on microgliosis (microglial proliferation), we first attempted to perform quantitative morphometry to assess Iba-1^+^ microgliosis over the time course. However, we found certain technical challenges to acquire accurate cell counts when a majority of Iba-1+ cells overlap in the ischemic hemispheres from one to four weeks after stroke [31]. Alternatively, we used the mean staining density of Iba-1 immunofluorescence to quantify the activation of microglia. Since Iba-1 is specifically expressed in microglia/macrophages and is up-regulated during the activation of these cells in ischemic stroke brain [31,32], we reasoned that the density of Iba-1 immunofluorescence proportionally reflects both Iba-1+ cell number and level of Iba-1 protein in microglia/macrophages, which is sufficient to represent the changes of microgliosis. Rats were killed and perfused for vessel counting at 24 hours, 48 hours, and one, two and four weeks of reperfusion after stroke. Eight brain sections from each animal with an interval of 100 μm covered a span of 800 μm in the peri-Bregmal region (approximately 1.00 to 0.2 mm rostral to Bregma) [3]. Microglia labeled with Iba-1 were measured in images captured from ischemic hemispheres with a low power objective lens (10×) by using ImageJ (National Institutes of Health, Bethesda, MD, USA). Indicators of animal identity on slides were blinded to the investigator. Three images from the ischemic area were captured for each section. The intensity of Iba-1 fluorescence was calculated as the mean of the intensity obtained from the imaged sections.

**Figure 1:**
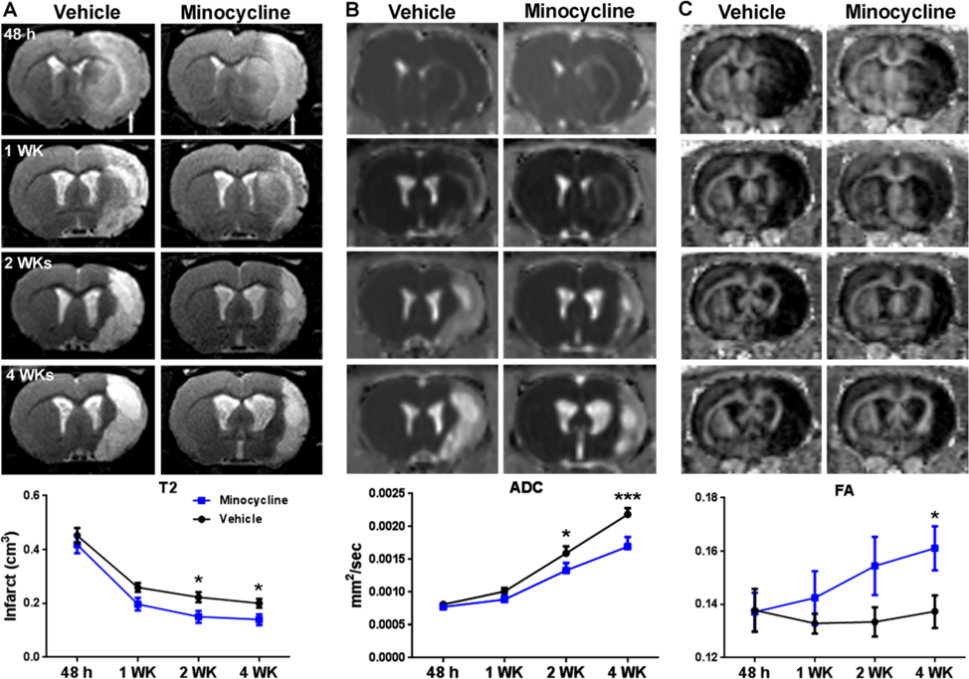
**Stroke recovery monitored by magnetic resonance imaging. A**. Anatomical T2 MR images. Arrows indicate ischemic hemispheres. Line graph demonstrates quantification of infarct volumes in ischemic hemispheres. **B**. ADC maps. Line graph demonstrates quantification of edema (acute stage) and tissue loss (late stage) in ischemic hemispheres. **C**. FA maps. Line graph demonstrates quantification of white matter change in ischemic hemispheres. *P <0.05, ***P <0.001 versus vehicle group, n = 8 in each group.

For analysis and quantification of cytokines with Iba-1, eight brain sections from each animal were used as described above. Three areas from ischemic hemispheres were captured with a 40× objective lens. Automatic and quantitative measurement of co-localization of cytokines and Iba-1 was performed using co-localization plugin Coloc 2 in Fiji-ImageJ (National Institutes of Health, Bethesda, MD, USA). Briefly, two color channel images were converted into 8-bit gray images. After background subtraction, co-localization analysis was done in Coloc 2. A two-dimensional intensity histogram, which displayed the correlation between the intensities of the two color channels, was presented. Among multiple quantitative results, we chose to report Li *et al*.’s intensity correlation quotient (ICQ), which provides an overall index of co-localization. The ICQ values are distributed between −0.5 and +0.5. Random staining: ICQ approximately 0; segregated staining: 0 > ICQ −0.5; co-localized staining: 0 < ICQ ≤ +0.5 [33].

### Statistical analysis

Unpaired t-tests or one-way Analysis of Variance (ANOVA) were performed for two groups or for multiple group comparisons (with a *post-hoc* Student-Newman-Keuls test), respectively. Two-way (−factors) ANOVA were performed for group comparisons with time course analysis. In all statistical tests, differences were considered significant when *P* <0.05. Data are presented as means + SE. Statistical analysis was performed using Prism, version 6.0 (GraphPad Software Incorporated, La Jolla, CA, USA).

## Results

### Minocycline significantly improved stroke outcome during recovery

By taking advantage of MRI, we temporally measured lesion sizes in ischemic hemispheres (Figure 1A). Compared with the vehicle group, T2-weighted images showed significantly smaller lesions in the minocycline group at two weeks that continued to four weeks. A decrease in the ADC value was found in the ischemic hemispheres at two and four weeks, suggesting decreased diffusivity in the regions of tissue damage (Figure 1B). In line with the anatomic T2 images, the ADC maps demonstrated significantly reduced tissue breakdown in the minocycline group at two and four weeks after stroke. Compared with the vehicle group, FA [34] in the minocycline group (Figure 1C) showed white matter that was significantly protected from damage at four weeks.

**Figure 2:**
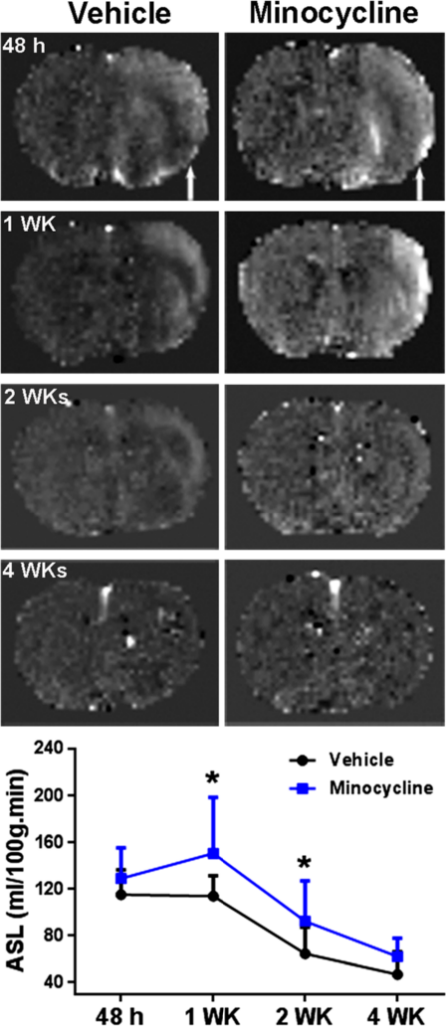
**ASL maps monitored by MRI at 48 hours and one, two and four weeks after stroke.** Arrows indicate ischemic hemispheres. Line graph demonstrates quantification of blood flow in peri-infarct areas. *P <0.05 versus vehicle group, n = 8 in each group.

### Minocycline facilitates cerebral blood flow and reduces blood–brain barrier permeability in peri-infarct areas

**Figure 3:**
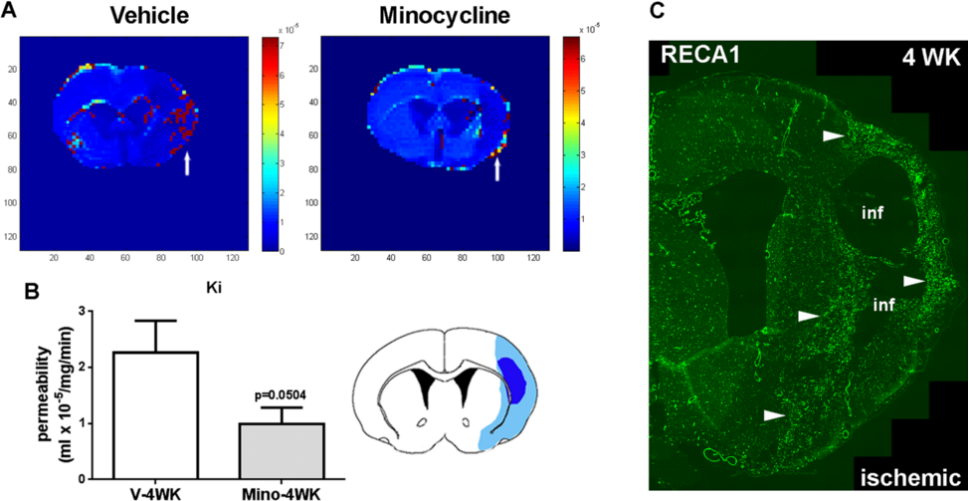
**Blood–brain barrier permeability monitored by magnetic resonance imaging. A**. Parametric image *K*_i_ map by DCE-MRI represents BBB transfer rate at four weeks after stroke. Color-coded permeability coefficient maps reconstructed from contrast-enhanced MRI data demonstrate the regions of high (red) and low (blue) permeability. **B**. Histogram demonstrates the quantification of BBB permeability in peri-infarct areas (light blue areas in the brain slice cartoon). *P = 0.0504 versus vehicle group, n = 8 in each group. **C**. RECA1 (marker of endothelial cells) immunostaining shows increased density of new vessels in the peri-infarct area (arrows), corresponding to the regions where BBB transfer rate and plasma volume were measured.

We measured the CBF with ASL (Figure 2). CBF maps showed hyperperfusion in the lesion hemisphere, specifically in the peri-infarct areas, at 48 hours and one week compared with the contralateral normal hemispheres, as seen previously [35]. We found that the hyperperfusion in the lesion hemisphere began to reduce after two weeks. However, higher perfusion still can be seen in the peri-infarct areas at two weeks. This observation was also seen in ischemic brains at three weeks reperfusion in our previous study, which correlates to the high density of newly formed vessels in the peri-infarct areas [3]. The graph in Figure 3 shows that minocycline caused a higher ASL value at each time point. Significant differences between the vehicle and minocycline groups were seen at one and two weeks.

Increased BBB permeability in the angiogenic vessels was seen due to immature BBB functions with a lack of some major TJPs [3]. DCE-MRI showed a remarkably decreased BBB transfer rate (*K*_i_) in the peri-infarct area in the minocycline group at four weeks compared with the vehicle group (Figure 3A,B). The light blue area in the brain cartoon of Figure 3B indicates the region where the BBB permeability of angiogenic vessels [3] in peri-infarct areas (Figure 3C) was measured.

**Figure 4:**
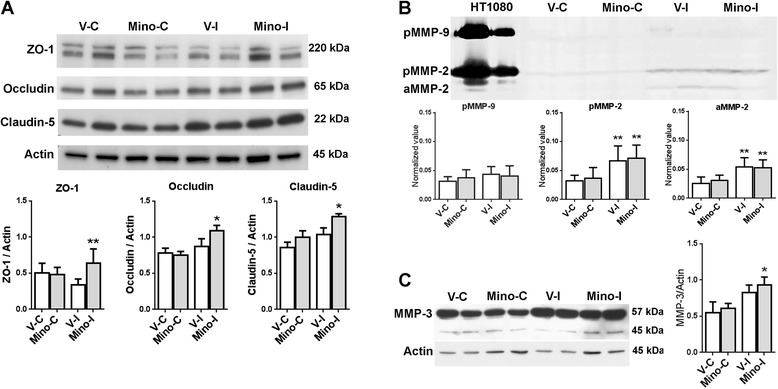
**Expression of tight junction proteins and matrix metalloproteinases at four weeks after stroke. A**. Western blot analysis for TJPs. ZO-1: **P <0.05 versus V-I, V-C and Mino-C. Occludin: *P <0.05 versus V-I, V-C and Mino-C. Claudin-5: *P <0.05 versus V-I, V-C and Mino-C. **B**. Gel zymography analysis for MMP-2 and −9. **P <0.01 versus V-C and Mino-C. n = 8 in the vehicle group, 9 in the minocycline group. **C**. Western blot analysis for MMP-3. Level of MMP-3 including preform (57 kDa) and active form (45 kDa): *P <0.05 versus V-C and Mino-C. Mino-C: minocycline contralateral. Mino-I: minocycline ipsilateral. V-C: Vehicle contralateral. V-I: Vehicle ipsilateral. n = 8 in the vehicle group, 9 in the minocycline group.

### Minocycline enhances levels of tight junction proteins and matrix metalloproteinase-3 during recovery

Western blot analysis revealed that the levels of TJPs, including (zona occluden-1) ZO-1, occludin and claudin-5, in the ischemic cortices of minocycline-treated rats were significantly higher compared with the vehicle-treated group (Figure 4A). Figure 4B shows that significantly increased levels of pro and active MMP-2 were detected in both vehicle- and minocycline-treated ipsilateral cortices compared with the contralateral, while no differences were detected between the vehicle and minocycline groups. A very low expression of MMP-9 was detected in ischemic brains at four weeks, consistent with our previous observation [3]. Increased levels of MMP-3 were seen both in vehicle- and minocycline-treated ipsilateral cortices (Figure 4C). A significant difference was only detected between the contralateral and ipsilateral cortex in minocycline-treated brains. As expected, all treatments had no significant effect on the basal levels of TJPs and MMPs in the contralateral tissue.

**Figure 5:**
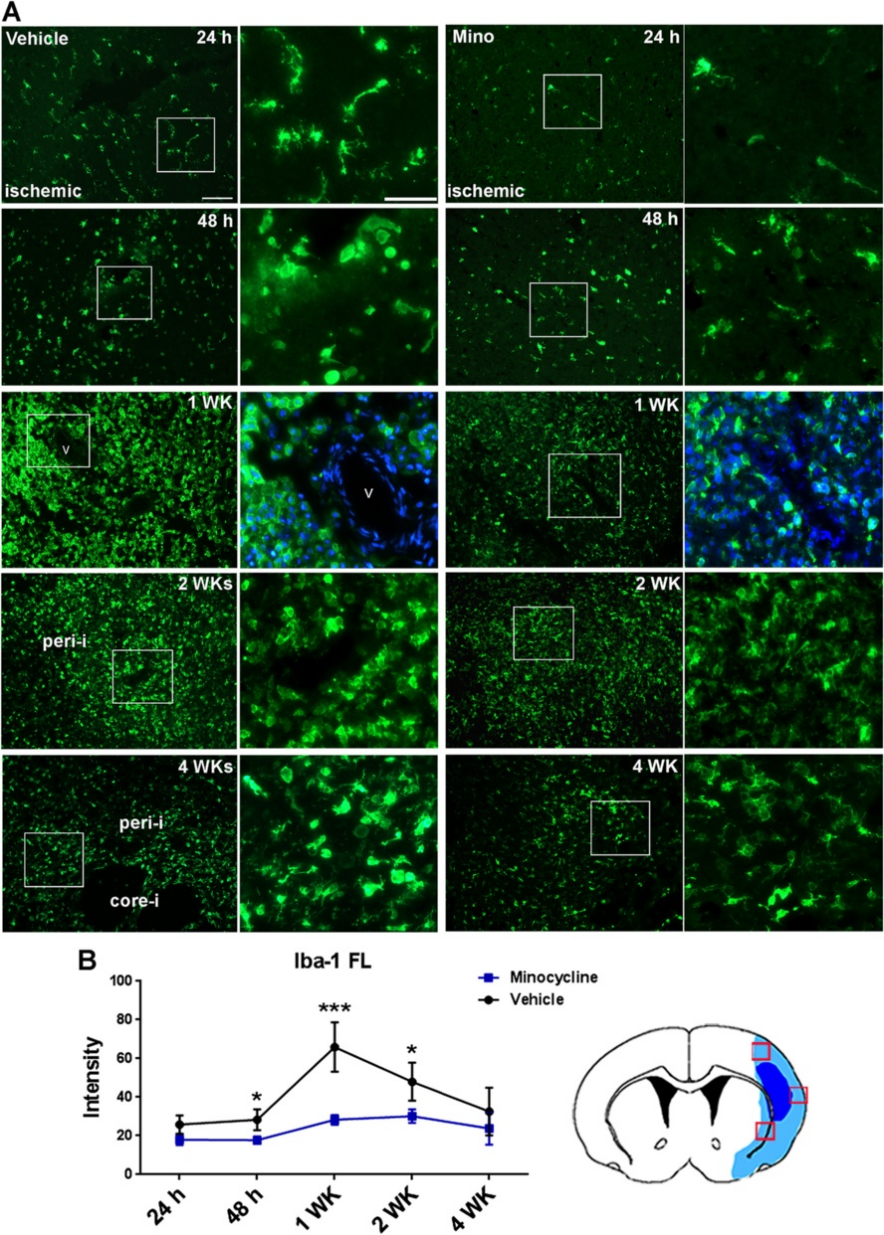
**Microglia/macrophage activation by immunofluorescence staining of Iba-1. A**. Morphological changes of microglia/macrophage in ischemic hemispheres over reperfusion courses. Scale bar = 100 μm. The right panels at each time point present a higher magnification of the images shown in squares in the left panels. Scale bar = 50 μm. DAPI was used to show nuclei and vessel at one week. core-i: core infarct area; peri-i: peri-infarct area; V: vessel. **B**. Quantification of Iba-1 fluorescence (FL) intensity in ischemic hemispheres over reperfusion courses. *P <0.05, ***P <0.001 versus vehicle group, n = 5 in each group. The brain cartoon shows the three images measured by ImageJ that were obtained from the infarct areas, indicated by the squares in red.

### Inflammatory cytokines expressed by alternatively activated microglia/macrophages are involved in neurovascular remodeling

To evaluate the effect of minocycline on the alternative activation of microglia activation, we next investigated the response of microglia to stroke and reperfusion injury by using immunohistochemistry with an antibody against Iba-1. Iba-1 is a 17-kDa protein that is specifically expressed in microglia and macrophages and is up-regulated during the activation of these cells [31,32]. Microglia and macrophages are virtually undistinguishable since they express the most commonly used cell markers *in vivo*. Fortunately, recent data show that the vast majority of Iba-1-positive cells in the ischemic brain represent activated resident microglia [20,36,37]. However, its specificity for microglia staining is limited in injured brain tissue, where peripheral macrophages may infiltrate [32]. Therefore, the Iba-1- positive cells were referred to as microglia/macrophages. Additional file 1: Figure S1 shows that Iba-1-positive microglia/macrophages were seen in ischemic hemispheres as early as 24 hours after reperfusion and reached a peak at one week that extended to four weeks. Various morphological shapes of microglia/macrophages were seen at different time points (Figure 5A). Microglia/macrophages with extending processes were the majority of Iba-1-positive cells in the ischemic hemispheres at 24 hours, which exhibited morphological evidence of activation [38]. At 48 hours, round Iba-1-positive microglia/macrophages began to appear. By one week, the vast majority of Iba-1-positive cells in the ischemic brain were round, especially in the core infarct areas. However, microglia/macrophages with extending processes reappeared in the peri-infarct areas by two weeks, and were continually seen for up to four weeks (Figure 5A and Additional file 1: Figure S1).

**Figure 6:**
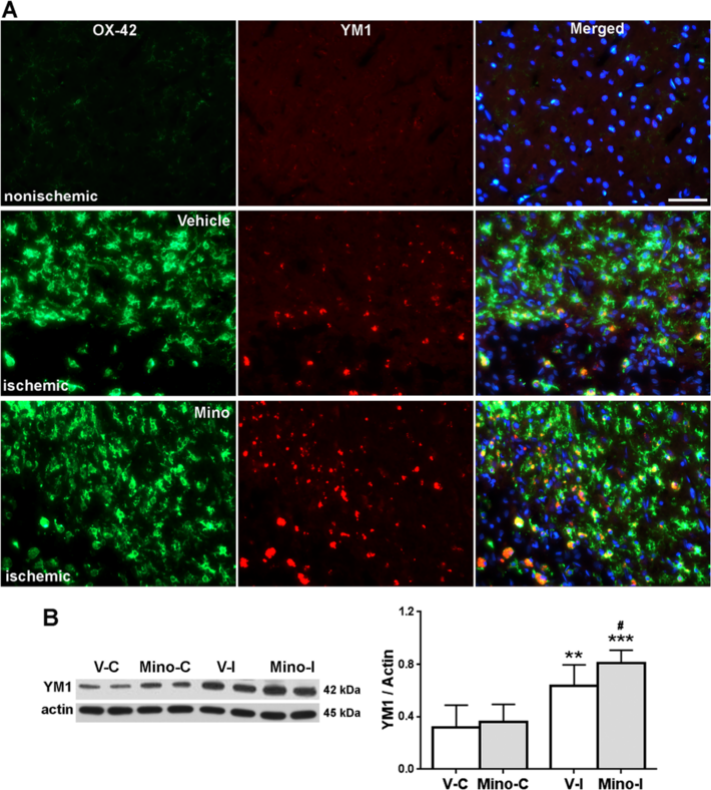
**Phenotype of microglia/macrophage alternative activation at four weeks after stroke. A**. Double immunostaining shows expression of YM1 in microglia/macrophage (OX-42). Scale bars = 50. DAPI was used to show nuclei. **B**. Western blot analysis for protein level of YM1. **P <0.01 versus V-C and Mino-C. ***P <0.001 versus V-C and Mino-C. ^#^ *P* <0.05 versus V-I. n = 8 in the vehicle group, 9 in the minocycline group.

A significant reduction of Iba-1 fluorescence intensity was detected from 48 hours to two weeks in the minocycline group compared with the vehicle (Figure 5B), suggesting that treatment with minocycline significantly decreased microglia/macrophage activation. We did not detect a significant reduction of Iba-1 fluorescence intensity in minocycline animals at four weeks. One of the reasons for this could be the greater tissue loss in ischemic hemispheres in the vehicle animals (Figures 1 and 5A and Additional file 1: Figure S1). Expression of M2 microglia/macrophage marker YM1 was co-localized in the microglia/macrophages (Additional file 1: Figure S2), suggesting the shifting of microglia/macrophage activation from M1 to M2 at four weeks. More importantly, IHC and Western blot analysis also showed that treatment with minocycline significantly increased expression of YM1 compared with vehicle treatment (Figure 6A,B). There were no detectable differences in YM1 levels in the contralateral tissues across the two animal groups.

**Figure 7:**
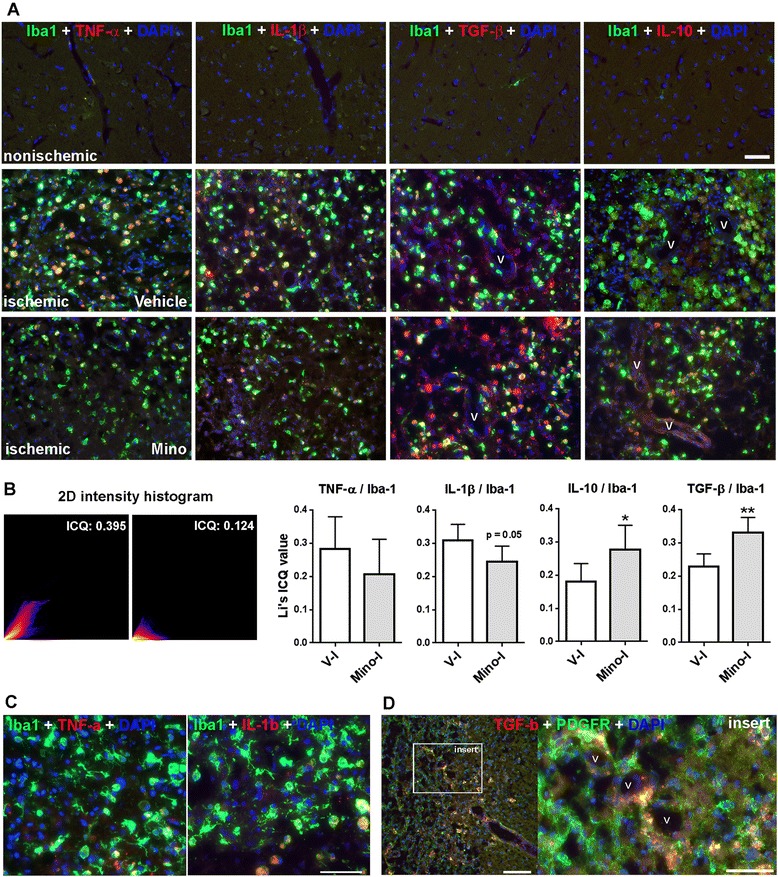
**Inflammatory factors expressed by active microglia/macrophage in peri-infarct areas at four weeks after stroke. A**. Double immunostaining shows expression of TNF-α, IL-1β, IL-10 and TGF-β in active microglia (Iba-1). **B**. Analysis and quantification for co-localization of cytokines and Iba-1 in the microglia/macrophages with Fiji-ImageJ. Representative two-dimensional histogram and scatterplots visualize the correlation of the pixel intensities, over all pixels and voxels in the images with different Li’s ICQ values, generated by Fiji-ImageJ. Statistical bar figures demonstrate the quantification of Li’s ICQ values for co-localization of each cytokine with Iba-1 in vehicle- and minocycline-treated ischemic hemispheres. *P <0.05, **P <0.01 versus V-I. n = 5 in each group. **C**. Double immunostaining shows little expression of TNF-α and IL-1β in active microglia/macrophage, with extending processes in the peri-infarct areas bordering with intact tissues. Scale bars = 50 μm. **D**. Double immunostaining shows expression of TGF-β in PDGFR-β-positive pericytes that closely surround the vessels (V). Scale bars = 50 and 100 μm.

We next investigated the expression of four cytokines in microglia/macrophages in peri-infarct areas at four weeks reperfusion: pro-inflammatory cytokines TNF-α and IL-1β, and anti-inflammatory cytokines TGF-β and IL-10. We found that the microglia/macrophages in the peri-infarct areas expressed all four cytokines (Figure 7A). However, minocycline reduced expression of TNF-α and IL-1β, and significantly increased expression of TGF-β and IL-10 in the microglia/macrophages (Figure 7A,B). Furthermore, we found that little TNF-α or IL-1β staining was seen in the microglia/macrophages with extending processes in peri-infarct areas that are adjacent to intact tissues (far from core infarct regions) (Figure 7C). Using an antibody for PDGFR-β for immunostaining (an established early marker of activated pericytes) we demonstrated that the proliferating pericytes, which closely surround the newly formed vessels in the peri-infarct areas and are involved in the formation of TJPs [3], expressed TGF-β (Figure 7D).

### Minocycline increased expression of active anti-inflammatory cytokines in ischemic hemispheres during recovery

**Figure 8:**
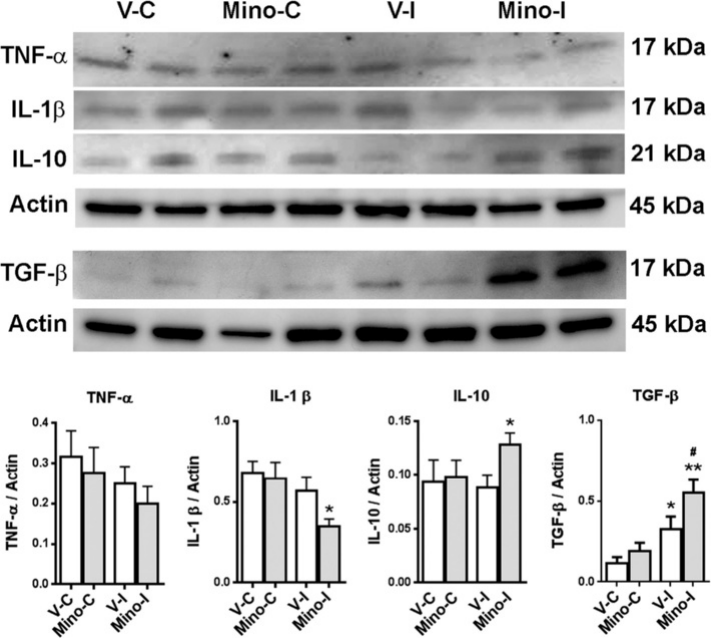
**Western blot analysis for active forms of inflammatory factors in rat brains at four weeks after stroke.** TNF-α: levels of TNF-α were decreased in ischemic rat brains and treatment with minocycline facilitated the reduction. IL-1β: *P <0.05 versus V-I. IL-10: *P <0.05 versus V-I. TGF-α: *P <0.05 versus V-I; ^##^ *P* <0.01 versus V-C and Mino-C. n = 8 in the vehicle group, 9 in the minocycline group.

Levels of active (cleaved) forms of these cytokines in the cortex at four weeks were assessed using Western blots (Figure 8). A lower amount of TNF-α and IL-1β in the ischemic cortex were seen compared with the contralateral cortex. Treatment with minocycline resulted in a significant reduction of IL-1β in the ischemic cortex compared with the vehicle. The levels of IL-10 and TGF-β in the ischemic cortices of minocycline-treated rats were significantly higher compared with the vehicle-treated group. The administration of minocycline significantly facilitated the expression of TGF-β in the ischemic cortex. As expected, minocycline had no significant effects on the levels of these cytokines in the contralateral tissues compared with the vehicle.

## Discussion

We demonstrate that a single dose of minocycline at an early stage of reperfusion after stroke effectively prevented brain damage, and impacted neurovascular remodeling favorably during recovery. This facilitated vascular remodeling in peri-infarct areas by promoting TJP formation and BBB function during recovery. In contrast, treatment with minocycline did not impair expression of MMP-2 and −3 during recovery, which are required for stroke- induced angiogenesis. Finally, we showed that minocycline promoted neuroprotective phenotype alternative activation of microglia/macrophages, which is involved in the neurovascular remodeling after stroke.

Minocycline has shown anti-inflammatory, anti-apoptotic and neuroprotective effects, as well as inhibition of MMPs, in many models of cerebral ischemia and neurodegenerative disease. High penetration of the BBB and a good safety profile make minocycline ideal for use in stroke treatment [4,6]. However, prior studies of minocycline in animal models have investigated neuroprotective effects during acute and subacute stages [4,11], and the late effects on neurovascular remodeling are less studied. By taking advantage of non-invasive MRI, we monitored the outcome during spontaneous and minocycline-induced recovery. Multimodal MRI analysis with anatomical T2, ADC and FA showed significant protection from brain injury with minocycline from two to four weeks after stroke. The early neuroprotective role of minocycline on BBB may attenuate the secondary injury, such as entrance of neurotoxic blood compounds, infiltration of inflammatory cells, systemic MMPs and especially neutrophil-derived MMP-9 [39,40]. This would provide the cellular and molecular basis to promote later recovery from stroke. Considering the limited treatment options for ischemic stroke and the promising results of both early phase human trials and animal models, a drug like minocycline, producing even modest improvements in outcome, may substantially increase patient functional recovery and quality of life while decreasing public financial burden.

We noted that the significant improvements measured by MRI were first seen at two weeks after stroke, while significantly reduced infarct size minocycline treatment was detected at 48 hours after stroke by triphenyl tetrazolium chloride (TTC) staining of fresh rat brain tissues [11]. This may have resulted from MRI signals from penumbra areas, as well as postischemic hyperperfusion (PIH) [35]; while TTC stains only dead cells. In addition, present data show that a significant reduction of microglia activation by minocycline was seen beginning at 48 hours, consistent with the TTC stains. These differences between MRI in living subjects and histology in postmortem tissues seen in this study may provide important preclinical information for diagnosis and treatment for stroke.

PIH is thought to arise as a result of disturbed cerebral autoregulation. PIH has been reported to be harmful (aggravate edema and hemorrhage, and neuronal damage from reperfusion injury) and beneficial (prevent infarct growth) [35,41,42]. Our ASL data show that significantly higher CBF in peri-infarct regions with minocycline occurred from one to two weeks after reperfusion. Considering its protective role in BBB disruption, the higher perfusion in peri-infarct regions in response to minocycline treatment at a later stage is beneficial for repair. With magnetic resonance angiography (MRA), Tanaka *et al*. revealed recanalization of the right middle cerebral artery at two hours after stroke. The MRA at 48 hours also showed many additional dilated vessels in the affected hemisphere, which were highly perfused [35]. We proposed that minocycline reduced BBB damage and protected the vessels in lesion hemispheres at the acute stage, which increased the perfusion and prevented the reperfusion injury growth at the subacute stage that facilitates repair.

In this study, permeability coefficient maps (*K*_i_) demonstrate lower BBB permeability in minocycline-treated rats at four weeks, which was correlated with a higher blood flow (ASL) in peri-infarct regions, suggesting optimal barrier function. In line with the MRI results, treatment with minocycline enhanced the level of TJPs, claudin-5, occludin and ZO-1. TJPs are normally seen within endothelial cells, where they form the first barrier to blood-borne substances. Previously, we observed BBB leakage in the peri-infarct regions where new angiogenic vessels are located, indicating an incomplete barrier function due to absence of major TJPs, occludin and ZO-1 in endothelium. However, this endothelial leakage may be compensated, at least in part, by the overexpression of TJPs in pericytes and astrocytes that closely surround the new vessels [3], which contribute to extra-endothelial TJP formation during neurovascular remodeling. Our present data suggest the possibility that an enhanced restructuring of the BBB TJPs by minocycline may provide functionality to the BBB, in spite of an immature endothelium during neurovascular remodeling.

Stroke induces ischemic infarct with a cascade of metabolic and inflammatory consequences that extend into the penumbra. After stroke, microglia, the brain-resident macrophage [13], become activated, obtain an amoeboid morphology and release inflammatory cytokines (classical activation/the M1 phenotype) [20]. However, microglia can also be alternatively activated, performing crucial roles in limiting inflammation and phagocytosing tissue debris (alternative activation/the M2 phenotype) [13,18,20,36,43]. Studies reveal that the dynamics of the microglial immunophenotype change over time after ischemic stroke and that the location of the microglia (core versus penumbra) is critical in determining that phenotype [20]. Treatment with minocycline after stroke significantly reduced microglial activation in brains [20,44-46]. We postulated that minocycline inhibiting inflammatory response at an early stage of stroke may influence the microglia/macrophage immunophenotype change. In this study, we found various morphological shapes and locations (core infarct and peri-infarct) of microglia/macrophages at different time points over the four weeks of reperfusion. During the first few days, active microglia/macrophages, with extending processes located within the ischemic attacked regions, seemed to represent the brain resident microglia that rapidly responded to injury, which is comparable with other reports [31,37]. Morphological changes of active microglia/macrophages over time are similar with the time-dependent recruitment of brain resident microglia and blood-derived macrophages after stroke [31,37]. From two to four weeks, active microglia/macrophages with extending processes reappeared in the peri-infarct areas, especially in the areas adjacent to intact tissue, indicating a new population of active microglia/macrophages. The expression of M2 microglia/macrophage marker YM1 was co-localized in these active morphometry, suggesting shifting of microglia/macrophage activation from M1 to M2 at four weeks.

In cerebral ischemia, early research showed the effectiveness of minocycline in decreasing infarct size and inflammation when administered within five hours of an ischemic event [6,25]. Some early phase clinical trials have shown minocycline to be safe and potentially effective in acute ischemic stroke, alone or in combination with tissue plasminogen activator [4,7]. Our acute study on rat MCAO shows that minocycline alone or along with normobaric hyperoxia effectively reduces brain injury in transient focal cerebral ischemia, with protection due to the inhibition of MMP-2/9-mediated occludin degradation and attenuation of caspase-dependent and independent apoptotic pathways [11]. We postulated that minocycline inhibiting inflammatory response at an early stage of stroke may influence the microglia/macrophage immunophenotype change. The present data demonstrated that a single dose of minocycline treatment right after reperfusion onset induced significant inhibition of microglia/macrophage activation at 48 hours and increased YM1 expression in ischemic brains at four weeks following stroke. These results indicate that early treatment of minocycline after stroke that protects the BBB from disruption and attenuates inflammation, also sustains the ischemic tissue in brain, which facilitates the neuroprotective phenotype alternative activation of microglia/macrophage during the recovery stage.

Recent studies show that minocycline for five and seven days of treatment is able to attenuate infarct size and microglial activation induced by stroke by seven days [16] and by 22 days, respectively [17]. Because microglia are involved in neurogenesis and neuroplasticity following ischemic insult, inhibition of microglial activation is unlikely to be the sole mechanism of minocycline’s long-term neuroprotective effect. Continuous behavioral and neurological recoveries are seen when minocycline is administered four days following MCAO, and subsequently for four weeks [44,47]. In our study, at four weeks, the active microglia/macrophages surrounding and within the peri-infarct areas expressed both pro-inflammatory factors (TNF-α and IL-1β) and anti-inflammatory factors (TGF-β and IL-10) [36]. Further, reduced TNF-α and IL-1β were seen in the microglia/macrophages with extended processes that were seen in the peri-infarct border adjacent to intact tissues, suggesting a change in phenotype depending on the source and active timing of microglia/macrophages [13,20]. Importantly, our current data show that one dose of minocycline treatment at an early stage of reperfusion promotes the neuroprotective phenotype alternative activation of microglia/macrophage during recovery, and increased expression of TGF-β and IL-10. TGF-β and IL-10 are pleiotropic immunoregulatory cytokines that have a crucial role in the development of the anti-inflammatory milieu associated with tissue repair [36]. Although TGF-β is well known for its pro-inflammatory effects, it can also suppress inflammation by inhibiting T helper (TH) type 1 and TH2 responses and promoting Treg cell development. Similarly, the immunoregulatory cytokine IL-10, produced by multiple cell types including Treg cells, has both neuroprotective and anti-inflammatory activities [36].

Besides the active microglia/macrophages, the proliferating pericytes, closely surrounding angiogenic vessels in the peri-infarct areas, also expressed TGF-β. We and others have shown that the proliferating vascular pericytes acquire a microglial phenotype after stroke by expressing microglial markers, such as NG2, Gal-3, Iba-1 and CD11b [3,48]. The TGF-β signaling pathway has been demonstrated to be involved in the regulation of BBB functional integrity and TJP expression during inflammation, and may lower the endothelial permeability [49,50]. We previously showed that the microglia-like pericytes participate in the neovascularization of peri-infarct areas after stroke by expressing TJPs [3]. Our data suggest a novel treatment potential for facilitating neurological recovery by influencing these spontaneous repair-related alternative activation of microglia/macrophages in the brain after stroke with medicine exposure.

## Conclusions

We demonstrated that a single dose of minocycline administered early after stroke benefits neurovascular remodeling during the recovery phase. Functional alternative activation of microglia is involved in the BBB restoration during neurovascular remodeling after stroke. We found that minocycline promotes the neuroprotective phenotype of microglia/macrophage alternative activation during recovery. Due to the limited treatment time for acute stage in ischemic stroke, the potential acute and delayed effects of therapeutic intervention with minocycline will provide important preclinical information with strong translational potential.

## Additional file

Additional file 1: Figure S1. Immunofluorescence photomicrographs representing Iba-1-positive microglia/macrophages in ischemic hemispheres at different time points after stroke. Arrows indicate ischemic hemisphere. **Figure S2.** Double-immunofluorescence staining represented expression of M2 microglia/macrophage marker YM1 in active microglia/macrophage (OX-42) in ischemic hemispheres at four weeks after stroke. Peri-I: peri-infarct area. Scale bars = 100 μm.

## Abbreviations

ADC: apparent diffusion coefficient
ANOVA: analysis of variance
ASL: arterial spin labeling
BBB: blood–brain barrier
CBF: cerebral blood flow
DCE-MRI: dynamic contrast-enhanced-MRI
DMSO: dimethyl sulfoxide
EPI: echo-planar imaging
FA: fractional anisotropy
FAIR-RARE: flow-sensitive alternating inversion recovery rapid acquisition with relaxation enhancement
Iba-1: ionized calcium biding adapter molecule 1
ICQ: intensity correlation quotient
IL-1β: interleukin-1β
IL-10: interleukin-10
ISA: image sequence analysis
MCAO: middle cerebral artery occlusion
MMP: matrix metalloproteinase
MRA: magnetic resonance angiography
MRI: magnetic resonance imaging
NBO: normobaric hyperoxia
PDGFR-β: beta-type platelet-derived growth factor receptor
PIH: postischemic hyperperfusion
PVDF: polyvinylidene fluoride
RECA1: rat endothelial cell antigen-1
SHR: spontaneously hypertensive rat
TGF-β: transforming growth factor-β
TJP: tight junction protein
TNF-α: factors tumor necrosis factors-α
TTC: triphenyl tetrazolium chloride
ZO-1: zona occluden-1
