# Supplementary material for: Attenuation of acute stroke injury in rat brain by minocycline promotes blood–brain barrier remodeling and alternative microglia/macrophage activation during recovery
Source: J Neuroinflammation. 2015 Feb 10;12:26. doi: 10.1186/s12974-015-0245-4 (PMC4340283; doi:10.1186/s12974-015-0245-4)
Supplement: Additional file 1: Figure S1. — Immunofluorescence photomicrographs representing Iba-1-positive microglia/macrophages in ischemic hemispheres at different time points after stroke. Arrows indicate ischemic hemisphere. Figure S2. Double-immunofluorescence staining represented expression of M2 microglia/macrophage marker YM1 in active microglia/macrophage (OX-42) in ischemic hemispheres at four weeks after stroke. Peri-I: peri-infarct area. Scale bars = 100 μm. [file 12974_2015_245_MOESM1_ESM.doc]

**Supplementary Figures and Figure Legends**


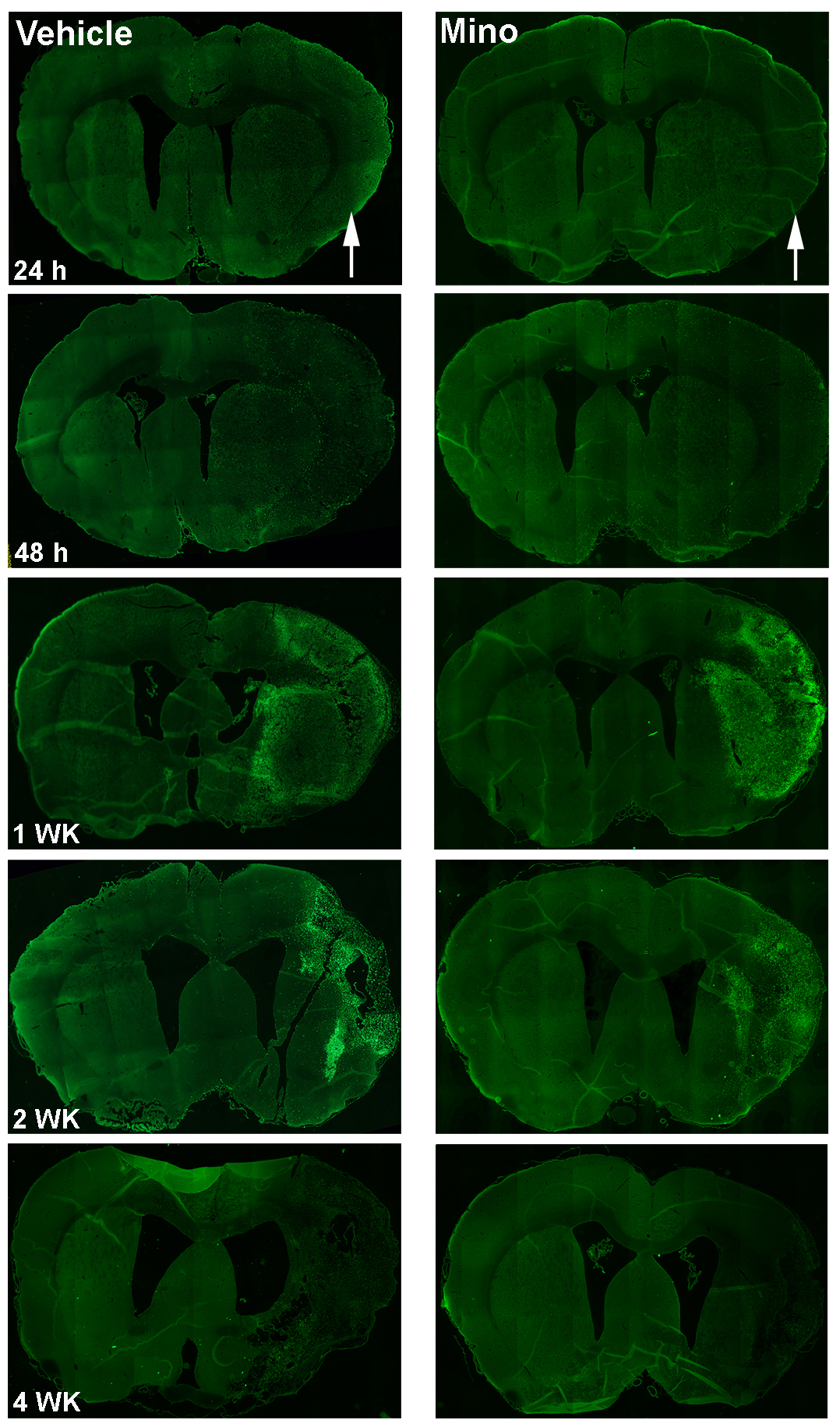


**Supplementary Figure 1. Immunofluorescence photomicrographs representing Iba-1-possitive microglia/macrophages in ischemic hemispheres at different time points after stroke. Arrows indicate ischemic hemisphere.**


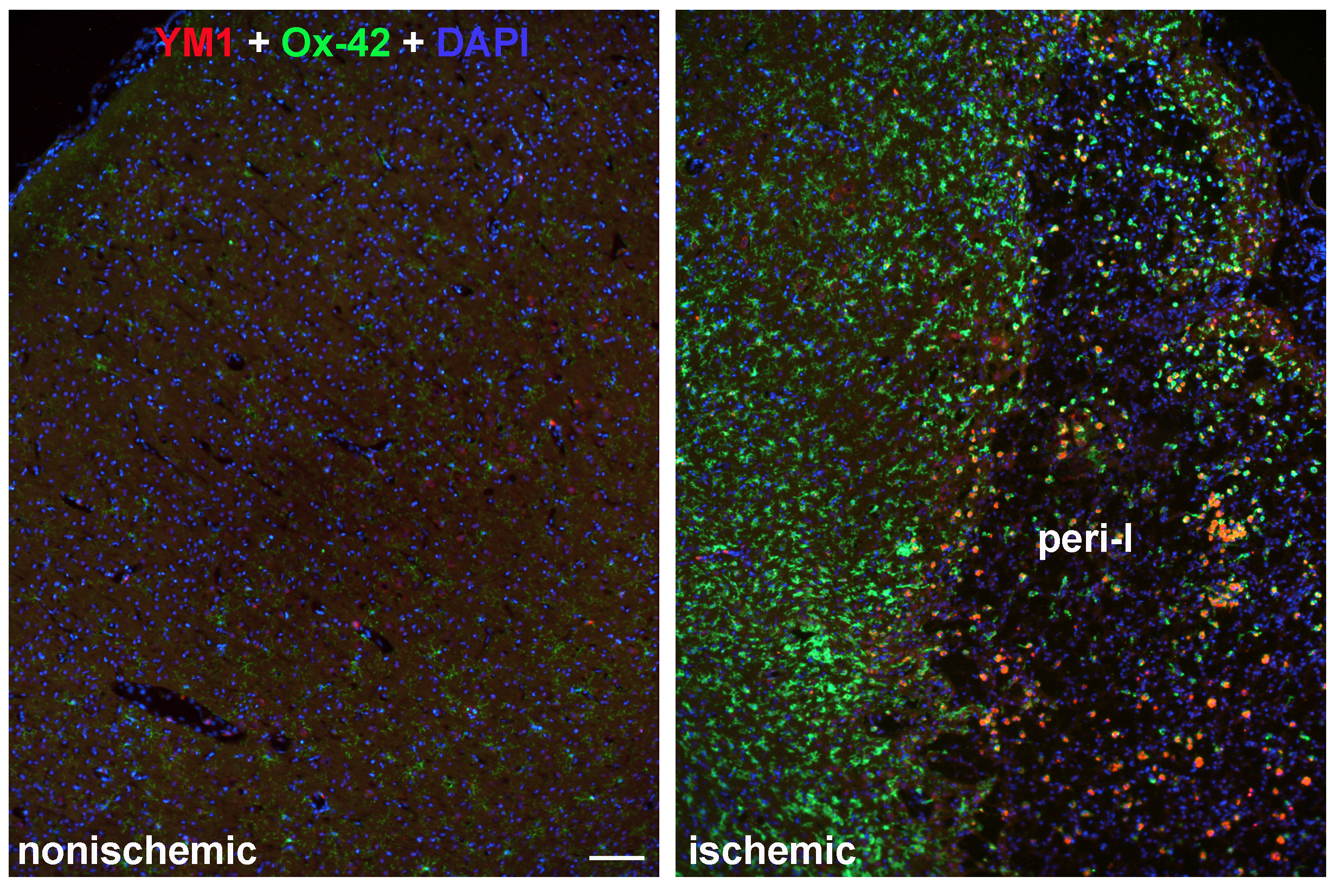


**Supplementary Figure 2. Double-immunofluorescent staining represented expression of M2 microglia/macrophage marker YM1 in active microglia/macrophage (Ox-42) in ischemic hemispheres at 4 weeks after stroke. Peri-I: peri-infarct area. Scale bars = 100µm.**
